# Synthesis, Antioxidant, and Antidiabetic Activities of Ketone Derivatives of Succinimide

**DOI:** 10.1155/2022/1445604

**Published:** 2022-03-28

**Authors:** Bushra Waheed, Syed Muhammad Mukarram Shah, Fida Hussain, Mohammad Ijaz Khan, Anwar Zeb, Muhammad Saeed Jan

**Affiliations:** Department of Pharmacy, University of Swabi, Swabi, KP, Pakistan

## Abstract

The prevalence of diabetes mellitus is persistently increasing globally creating a serious public health affliction. Diabetes mellitus is categorized into two major types designated as type I and Type II. Type I diabetes mellitus is characterized by complete lack of secretion of insulin, while Type II diabetes mellitus is the resistance of peripheral tissues to the action of insulin and inadequate compensatory secretion of insulin. Chronic hyperglycemia associated with diabetes causes failure of cardiovascular system, nervous system, kidneys, and eyes. At present, different types of drugs are used for the management of diabetes, but each of them is associated with more or less serious side effects. Therefore, we need to develop new therapeutic agents that have better efficacy and safety profile. In this study, three ketone derivatives of succinimides were synthesized based on Michael addition and characterized using NMR. All the synthesized compounds were checked for their *in vitro α*-amylase and *α*-glucosidase inhibitory activities. Further the synthesized compounds were also explored for their antioxidant activities, i.e, DPPH and ABTS assays. Based on the *in vitro* results, the synthesized compounds were further evaluated for *in vivo* antidiabetic activity. The synthesized compounds were (2-oxocyclohexyl)-1-phenylpyrrolidine-2,5-dione (BW1), benzyl-3-(2-oxocyclohexyl) pyrrolidine-2,5-dione (BW2), and (4-bromophenyl)-3-(2-oxocyclohexyl) pyrrolidine-2,5-dione (BW3). BW1 showed the highest inhibitory activity for DPPH causing 83.03 ± 0.48 at 500 *μ*g/ml with IC_50_ value of 10.84 *μ*g/ml and highest inhibitory activity for ABTS causing 78.35 ± 0.23 at 500 *μ*g/ml with IC_50_ value of 9.40 *μ*g/ml against ascorbic acid used as standard. BW1 also exhibited the highest activity against *α*-amylase and *α*-glucosidase inhibition causing 81.60 ± 0.00 at concentrations of 500 *μ*g/ml with IC_50_ value of 13.90 *μ*g/ml and 89.08 ± 1.04 at concentrations of 500 *μ*g/ml with IC_50_ value of 10.49 *μ*g/ml, respectively, against the standard drug acarbose.

## 1. Introduction

The prevalence of diabetes mellitus is persistently increasing globally creating a serious public health affliction [1]. It is predicted that the number of diabetic patients will increase from 171 a million in 2000 to 366 million by 2030 [2]. Diabetes mellitus is a group of metabolic diseases characterized by hyperglycemia arising from defects in insulin action, secretion, or both [3].

Based on etiopathogenesis, diabetes mellitus is categorized into two major types designated as type I and type II. Type-I diabetes mellitus is characterized by complete lack of secretion of insulin [4], while Type II diabetes mellitus (DM) caused by the resistance of peripheral tissues to the action of insulin and inadequate compensatory secretion of insulin [5]. Therefore, management of diabetes involves the reduction of postprandial hyperglycemia. Reduction of postprandial hyperglycemia can be obtained by inhibition of the carbohydrate-metabolizing enzymes. The *α*-amylase and *α*-glucosidase enzymes have major roles in carbohydrates metabolism. The *α*-amylase hydrolyzes complex carbohydrates, whereas *α*-glucosidase leads to the digestion of starch and disaccharides into glucose [6], therefore for the treatment of diabetes mellitus, *α*-amylase and *α*-glucosidase inhibitors may be used as potent compounds [7].

Complications of the diabetes mellitus include failure of cardiovascular system, nervous system, kidneys and eyes [8]. Microvascular complications include diabetic neuropathy, diabetic nephropathy [9] and diabetic retinopathy [10]. These complications of diabetes consist of enhanced arteriosclerosis of the arteries which supply blood to the heart, brain and lower parts of the body leading to myocardial infarction, stroke, peripheral vascular disease, and heart failure [11].

According to recent research, reactive oxygen species (ROS) are produced at a greater rate in diabetic patients, and it has been found that they are strongly implicated in the development of diabetes-associated complications [12]. On the other hand, there is evidence of abnormal response of antioxidant genes in hyperglycemia, causing a decrease in the expression of antioxidant enzymes. The increase in ROS causes damage especially in the postprandial level of glucose [13], as it is evident from experimental as well as clinical research that oxidative stress has a key role in the pathogenesis of complications associated with diabetes mellitus [14].

In this study, *in vitro* antioxidant activity of the synthesized ketone derivatives has been evaluated by DPPH [15] and ABTS radical scavenging assays [16].

Recent literature survey revealed that various classes of drugs like sulfonylureas, biguanides, thiazolidinediones, and dipeptidyl peptidase-4 (dpp-4) inhibitors are used for the management of diabetes but most of these are associated with obnoxious effects [17]. Therefore we need to develop new therapeutic moieties having better efficacy and safety profiles [18, 19].

Succinimide compounds contain an imide ring in their structures making them able to cross cell membranes easily inside the human body [20]. The compounds that cross the cell membrane can have access to the intracellular targets and *in vivo* activities. Ketone derivatives of succinimides have structural resemblance with that of thaizolidinedione (TZD). TZD is antidiabetic; therefore, it is presumed that ketones derivatives of succinimides have antidiabetic potential on the basis of its structure resemblance to that of TZDs. There is no literature on the antidiabetic potential of ketone derivatives of succinimides [21].

The aim of the current study was to explore the antioxidant and *in vitro* antidiabetic potential of the synthesized compounds. Furthermore, these compounds were also assessed in animal models.

## 2. Materials and Methods

Alloxan (CAS NO: 50-71-5), maleimides (CAS NO: 541-59-3), alpha-glucosidase (CAS NO: 9001-42-7), alpha-amylase (CAS NO: 9000-90-2), ethyl acetate (CAS NO: 141-78-6), n-hexane (CAS NO: 110-54-3), KOH (CAS NO: 1310-58-3), creatinine (CAS NO: 67-7-5), chloroform (CAS NO: 67-66-3), Tween 80 (CAS NO: 9005-65-6), and silica gel (CAS NO: 7631-86-9). Glibenclamide, phosphate buffer, and other required chemicals were purchased from the standard quality supplier.

### 2.1. Synthesis

Synthesis was carried out by reacting ketone species with maleimides using KOH and creatinine and chloroform as solvent. The reaction mixture was kept on the magnetic stirrer till completion of the reaction. TLC (thin layer chromatography) was used to check the completion of the reaction. After completion, the aqueous and organic layers were separated via separating funnel. This process was repeated thrice for proper separation. Rotary evaporator was used for solvent evaporation. Column chromatography was further used for the isolation of purified compounds [22].

### 2.2. Characterization

Various physical characteristics such as retardation factors and percent yields of the pure compounds were recorded. Structural details were elucidated by using ^1^H NMR and ^13^C NMR.

### 2.3. *In vitro* Assays

#### 2.3.1. ABTS Scavenging Activity

For the determination of the antioxidant activity of the synthesized compounds, the previously reported method [23] for ABTS scavenging activity was used with slight modification. The stock solution of 2.4 mM of potassium per sulfate solution and 7 mM ABTS solution was prepared by mixing them in equal volumes for 16 h. The stock solution thus prepared was diluted using methyl alcohol to give absorption of 0.7 ± 0.02 units when studied at the wavelength of 734 nm using a spectrophotometer. Fresh ABTS solution was prepared for each ABTS scavenging assay. About 150 *μ*L of the ABTS working solution was added to 50 *μ*L of synthesized compounds and kept in darkness for 10 min. The studies were repeated three times, and the results were demonstrated using mean ± standard deviation. Methanol was used as blank, and standard ascorbic acid was run at the same time. The absorbance was calculated utilizing a microplate reader at 734 nm.

#### 2.3.2. DPPH Scavenging Activity

The antioxidant assay of the synthesized compounds was conducted following the previously reported method [24]. A freshly prepared DPPH solution (50 *μ*L of 1.0 × 10^−3^ M) was added in methyl alcohol. Methanol was used as a control in this experiment. The prepared mixture was subjected to incubation at 25°C for 30 min. A decrease in the DPPH free radical concentration was calculated using a spectrophotometer at a wavelength of 517 nm. After incubation ascorbic acid was used as a positive control. The antioxidant activity was determined as a percent (%) of inhibition using the formula given:

(1) $$\begin{array}{c} \text{percent inhibition}=\frac{\text{absorbance}\left(\text{control}\right)-\text{absorbance}\left(\text{sample}\right)}{\text{absorbance}\left(\text{control}\right)}\times 100. \end{array}$$

#### 2.3.3. *α*-Glucosidase Activity

For *in vitro α*-glucosidase activity, samples were prepared by adding glucopyranoside to phosphate buffer solvent, and different concentrations of the synthesized compounds (31.25, 62.5, 125, 250, and 500 *µ*g/ml), respectively, were added to this solution and then the enzyme glucosidase in distilled water (0.5 *µ*g/ml) was also added to the above mixture. The reaction mixture thus prepared was subjected to incubation at 37°C for 20 minutes. The reaction was halted by adding HCl after incubation of the mixture. The intensity of the color was determined at 540 nm by spectrophotometer, and the formula given below was used for the determination of percent inhibition:

(2) $$\begin{array}{c} \text{percent inhibition}=\frac{\text{absorbance}\left(\text{control}\right)-\text{absorbance}\left(\text{sample}\right)}{\text{absorbance}\left(\text{control}\right)}\times 100. \end{array}$$

#### 2.3.4. *α*-amylase Activity

The *α*-amylase activity was studied following the previously documented protocol [25]. *α*-Amylase was added to phosphate buffer solvent, and the synthesized  compounds of different concentrations (31.25, 62.5, 125, 250, and 500 *µ*g/ml) were added in this solution. The starch solution was added, following incubation of the mixture, and the reaction mixture thus prepared was kept in water bath at 100°C for some time. Microplate reader measured the intensity of the color at 656 nm. Following formula was used to determine percentage inhibition:

(3) $$\begin{array}{c} \text{percent inhibition}=\frac{\text{absorbance}\left(\text{control}\right)-\text{absorbance}\left(\text{sample}\right)}{\text{absorbance}\left(\text{control}\right)}\times 100. \end{array}$$

### 2.4. *In Vivo* Studies

#### 2.4.1. Acute Toxicity Study

To establish the toxicity studies of the newly synthesized compounds, the experimental animals were divided into six groups, containing four animals in each group (*n* = 4). The synthesized compounds were administered at the dose of 200–1500 mg/kg body weight intraperitoneally (i.p). Animals were observed for 3 days after administration of the compounds for any unusual response [26].

#### 2.4.2. Animals Studies and Experimental Design

Alloxan was used for the induction of diabetes mellitus according to reported procedure [27]. Recently prepared Alloxan (ALX) was injected intraperitoneally to animals, fasting for 16 h, at a single dose of (150 mg/kg). Animals were observed for the development of diabetes by checking their glucose levels following the administration of ALX. Only diabetic animals, having random blood glucose level more than 200 mg/dl, were selected for studies. All the experimental procedure were approved from the ethical committee department of pharmacy, University of Swabi via letter No; UOS-06/2021.

A total of 30 animals were used to determine the hypoglycemic effect of the synthesized derivatives of succinimide. The experimental animals were segregated in six groups (*n* = 6), having five (5) animals in each group. Group I was designated as normal group and was given normal saline I/P only. Group II was given Tween 80, and Group III was given standard drug (glibenclamide) after the induction of diabetes. Group IV, Group V, and Group VI were given the various doses of synthesized compounds, respectively. The blood glucose level of each animal was recorded on 0^th^, 4^th^, 7^th^, and 15^th^ day of the experiment.

#### 2.4.3. Oral Glucose Tolerance Test (OGTT)

To perform OGTT mice fasted overnight were used including both control and treated mice. Glucose was administered orally at the dose of 2 g/kg against glibenclamide as standard drug. After administration of glucose, blood glucose level was calculated at time intervals of 0, 30, 60, and 120 min for assessment of the impact of exogenously given D-glucose on treated mice. OGTT was carried out 5 days before the completion of the study [28].

## 3. Results

### 3.1. Synthesis

#### 3.1.1. Procedure for the Synthesis of Ketone Derivatives of Succinimides

To the well-mixed solution of ketones (2 mmol) in chloroform, different N-substituted maleimides (1 mmol), creatinine, and 20 mol % KOH were added at room temperature. After the completion of the reaction, the reaction was quenched by adding a sufficient amount of water (15 ml). The chloroform portion was separated by using a separating funnel. The separation of the organic layer was repeated three times (each 15 ml). After separation, the organic layer was dried by low vacuum using rotary evaporator apparatus. The reaction mixture was then adsorbed at silica gel for loading into the column for purification. In column chromatography, *n*-hexane and ethyl acetate were used as a solvent. The yield of the final product was calculated from the pure product.

### 3.2. Characterization of the Synthesized Compounds

#### 3.2.1. (2-Oxocyclohexyl)-1-Phenylpyrrolidine-2,5-Dione (BW1)

**Figure 1:**
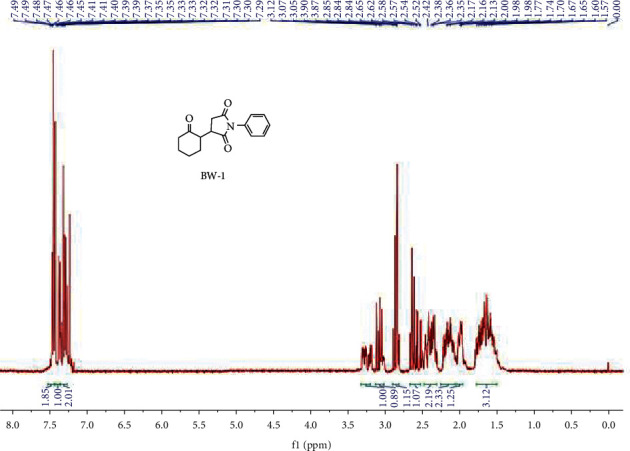
^1^HNMR spectrum of BW1.

**Figure 2:**
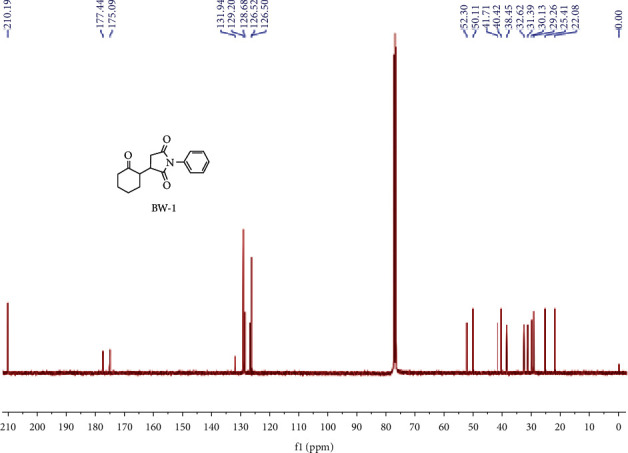
^13^CNMR spectrum of BW1.

White color product was obtained having 90 percent isolated yields, and the reaction was completed in 23 hours. A value of 0.41 was recorded as retardation factor using *n*-hexane and ethyl acetate in the ratio of 3 : 1, respectively. ^1^H NMR (400 MHz, CDCl_3_) (ppm): 7.44–7.50 (m, 2H). 7.35–7.40 (m, 1H), 0.24–7.33 (m, 2H), 3.19–3.33 (m, 1H), 3.02–3.12 (m, 1H), 2.82–2.90 (m, 1H), 2.52–2.67 (m, 1H), 2.31–2.48 (m, 2H), 2.07–2.22 (m, 2H), 1.96–2.04 (m, 1H), 1.51–1.82 (m, 3H) (Figure 1). ^13^C NMR (100 MHz, CDCl_3_) (ppm): 210.19, 177.44, 175.09, 131.94, 129.20, 128.68, 126.52, 126.50, 52.30, 50.11, 41.71, 40.42, 38.46, 32.62, 31.39, 30.13, 29.26, 25.41, 22.08,95.2 percent purity was displayed on HPLC analysis with retention time of 10 minutes, while LCMS analysis of C_16_H_17_NO_3_ is 272.2 [M + H] (m/z), and calculated yield is (%) N, 5.16, H, 6.32 & C, 70.83, while practical analysis (%) values for N: 5.21. H 6.29, and C: 71.03, Figure 2.

#### 3.2.2. Benzyl-3-(2-Oxocyclohexyl) Pyrrolidine-2,5-Dione (BW2)

**Figure 3:**
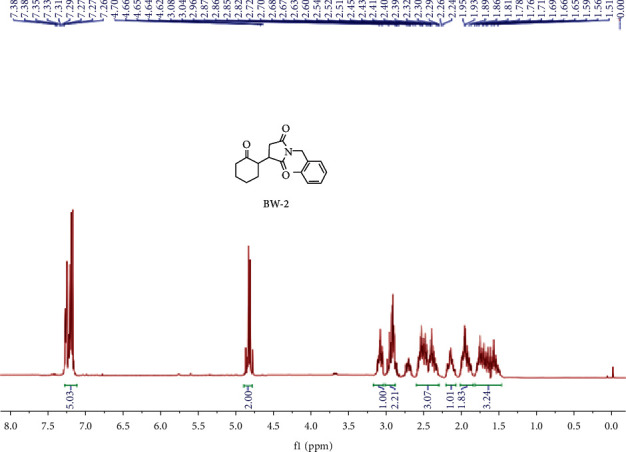
^1^HNMR spectrum of BW2.

**Figure 4:**
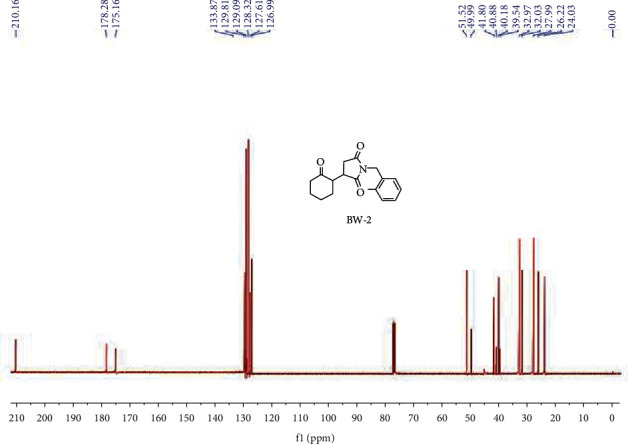
^13^CNMR spectrum of BW2.

*R*_f_ value was noted to be 0.45 for this compound in 3 : 1 *n*-hexane and ethyl acetate, respectively, with half white color, and 69 percent isolated yield while reaction was completed in 24 hours. ^1^H NMR (400 MHz, CDCl_3_) (ppm): 7.26–7.38 (m, 5H). (d, *J* = 7.38 Hz, 2H), 4.65, 3.02–3.16 (m, 1H), 2.80–2.99 (m, 2H), 2.33–2.63 (m, 3H), 2.12–2.26 (m, 1H), 1.83–2.01 (m, 2H), 1.49–1.79 (m, 3H), as shown in Figure 3. ^13^C NMR (100 MHz, CDCl_3_) (ppm): 210.16, 178.28, 175.16, 133.87, 129.81, 129.09, 128.32, 127.61, 126.99, 51.52, 49.99, 41.80, 40.88, 40.18, 39.54, 32.97, 32.03, 27.99, 26.22, 24.03, HPLC analysis showed 96.4% purity with 10.9 minutes retention time and LC-MS for C_17_H_19_NO_3_ is 286.2 [M + H] (m/z), alculated analysis is for N, 4.91; H, 6.71, and C is 71.56, and practical analysis is (%), N, 4.94. H, 6.73, and C 71.43, as shown in Figure 4.

#### 3.2.3. (4-Bromophenyl)-3-(2-Oxocyclohexyl) Pyrrolidine-2,5-Dione (BW3)

The reaction was completed in 30 hours with isolated yield of 75 percent. The color of the product was yellow. *R*_f_ value was recorded as 0.43 in 4 : 1 *n*-hexane and ethyl acetate, respectively.

**Figure 5:**
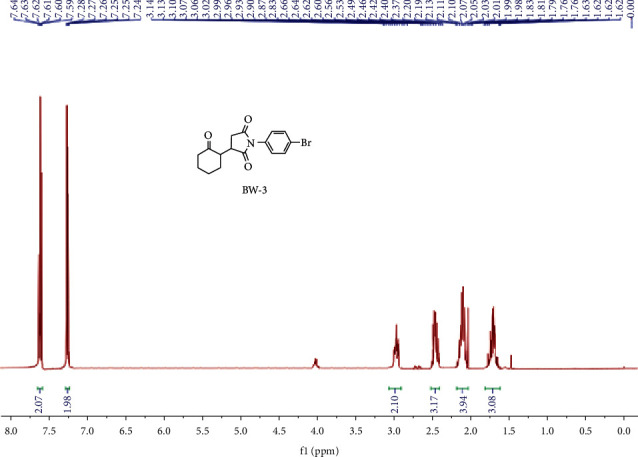
^1^HNMR spectrum of BW3.

**Figure 6:**
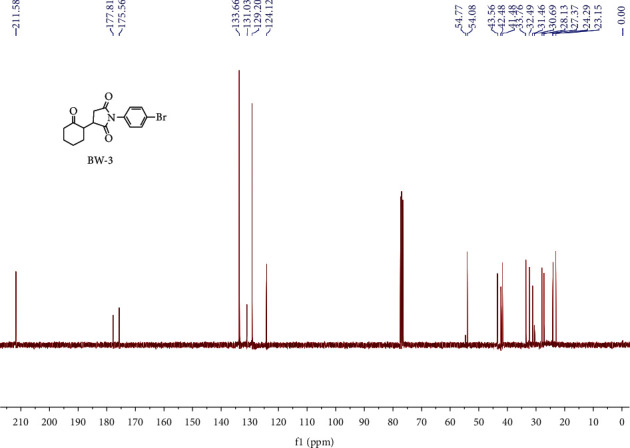
^13^CNMR spectrum of BW3.

^1^H NMR (400 MHz, CDCl_3_) (ppm): 7.59–7.64 (m, 2H), 7.24–7.28 (m, 2H), 1.96–3.02 (m, 2H), 2.47–2.52 (m, 3H), 2.06–2.21 (m, 4H), 1.64–1.77 (m, 3H), Figure 5. ^13^C NMR (100 MHz, CDCl_3_) (ppm): 211.58, 177.81, 175.56, 133.66, 131.03, 129.20, 124.12, 54.77, 54.08, 43.56, 42.48, 41.82, 33.76, 32.49, 31.46, 30.69, 28.13, 27.37, 24.29, 23.15 .98.1%, purity was displayed by the HPLC analysis with retention time of 13.9 minutes with LCMS value of 350.1 [M + H] (m/z) for C_16_H_16_BrNO_3_, while calculated analysis (%) N, 4.00, H, 4.61, and C is 54.87 and practical analysis of N, 4.03, H, 4.60, and C is 54.97 (%) Figure 6.

### 3.3. *In Vitro* Studies

#### 3.3.1. ABTS Scavenging Activity

In ABTS free radical scavenging activity, BW1 exhibited 78.35 ± 0.23, 73.36 ± 0.84, 70.62 ± 0.25, 66.16 ± 0.16, and 55.67 ± 0.32 percent inhibition at strengths 500, 250, 125, 62.50, and 31.25 *μ*g/ml, respectively, with IC_50_ value of 9.40 *μ*g/ml. BW2 exhibited 76.85 ± 2.24, 71.08 ± 0.47, 66.90 ± 0.96, 61.35 ± 0.51, and 57.40 ± 0.76 percent inhibition at strengths 500, 250, 125, 62.50, and 31.25 *μ*g/ml with IC_50_ value of 12.87 *μ*g/ml. BW3 exhibited 70.56 ± 1.06, 64.90 ± 0.45, 58.40 ± 0.82, 53.33 ± 0.66, and 48.42 ± 0.43 percent inhibition at strengths 500, 250, 125, 62.50, and 31.25 *μ*g/ml with IC_50_ value of 36.82 *μ*g/ml, the results are given in Table 1. Out of the three compounds investigated for antioxidant activity of ABTS BW1 showed the highest ABTS (9.40 *μ*g/ml) followed by BW2 (12.87 *μ*g/ml) and BW3 (36.82 *μ*g/ml)), respectively, against the standard ascorbic acid of IC_50_ value of 6.84.

#### 3.3.2. DPPH Scavenging Activity

In DPPH free radical scavenging test, the compound BW1 exhibited 85.83 ± 0.47, 77.23 ± 0.96, 72.29 ± 0.57, 67.33 ± 0.55, and 62.03 ± 0.77 percent inhibition at strengths 500, 250, 125, 62.50, and 31.25 *μ*g/ml, respectively, with IC_50_ value of 10.84 *μ*g/ml. BW2 exhibited 75.09 ± 0.32, 71.67 ± 1.20, 66.40 ± 0.25, 61.58 ± 1.12, and 56.65 ± 1.34 percent inhibition at strengths 500, 250, 125, 62.50, and 31.25 *μ*g/ml with IC_50_ value of 12.81 *μ*g/ml against ascorbic acid used as standard with IC_50_ value of 7.10 *μ*g/ml. BW3 exhibited 83.03 ± 0.48, 76.90 ± 0.48, 71.79 ± 0.63, 66.67 ± 0.61, and 57.69 ± 0.77 percent inhibition at strengths 500, 250, 125, 62.50, and 31.25 *μ*g/ml with IC_50_ value of 15.93 *μ*g/ml. BW1 showed highest inhibitory activity for DPPH free radical with IC_50_ value of 10.84 *μ*g/ml followed by BW2 with IC_50_ value of 12.81 *μ*g/ml, and BW3 value of 15.93 *μ*g/ml, respectively, against ascorbic acid used as standard with IC_50_ value of 7.10 *μ*g/ml. The results are given in Table 1.

#### 3.3.3. *α*-Glucosidase Activity

The inhibitory activities of BW1, BW2, and BW3 on alpha-amylase and alpha-glucosidase enzymes were explored in this study, and the results are presented in Table 2.

In the alpha-glucosidase inhibitory activity, BW1 showed percent inhibition of 89.08 ± 1.04, 83.45 ± 0.90, 76.58 ± 0.63, 70.40 ± 0.20, and 65.80 ± 0.90 at concentrations 500, 250, 125, 62.50, and 31.25 *µ*g/ml, respectively, with IC_50_ value of 10.49 *µ*g/ml. BW2 exhibited percent inhibition of 87.36 ± 0.49, 81.34 ± 0.55, 74.39 ± 0.49, 68.47 ± 0.52, and 62.44 ± 0.55 at concentrations 500, 250, 125, 62.50, and 31.25 *µ*g/ml, respectively, with IC_50_ value of 10.81 *µ*g/ml. BW3 displayed percent inhibitions of 77.85 ± 2.24, 72.08 ± 0.47, 67.90 ± 0.96, 63.28 ± 0.57, and 57.47 ± 0.56 at concentrations 500, 250, 125, 62.50, and 31.25 *µ*g/ml, respectively, with IC_50_ value of 12.15 against the standard acarbose of IC_50_ value of 8.20 *µ*g/ml.

#### 3.3.4. *α*-Amylase Activity

In the alpha-amylase inhibitory activity, BW1 showed percent inhibitions of 81.60 ± 0.00, 75.32 ± 0.40, 71.78 ± 0.44, 65.08 ± 0.66, and 57.40 ± 0.40 at concentrations of 500, 250, 125, 62.50, and 31.25 *µ*g/ml with IC_50_ value of 13.90 *µ*g/ml. BW2 exhibited percent inhibition of 78.69 ± 0.14, 73.14 ± 0.49, 67.44 ± 0.15, 63.72 ± 0.11, and 55.85 ± 0.17 at concentrations of 500, 250, 125, 62.50, and 31.25 *µ*g/ml with IC_50_ value of 15.34 *µ*g/ml. BW3 displayed percent inhibition of 75.35 ± 0.89, 71.36 ± 1.15, 67.62 ± 0.03, 62.16 ± 0.12, and 54.67 ± 0.35 at concentrations of 500, 250, 125, 62.50, and 31.25 *µ*g/ml, respectively, with IC_50_ value of 18.20 *µ*g/ml against the standard acarbose of IC_50_ value of 10.35 *µ*g/ml. The results are shown in Table 2, which indicate that out of three compounds, BW1 showed the highest alpha-glucosidase inhibition activity with IC_50_ value of 10.49 *µ*g/ml followed by BW2 with IC_50_ value of 10.81 *µ*g/ml and BW3 with IC_50_ value of 12.15 *µ*g/ml, respectively, and BW1 showed the highest alpha-amylase inhibition with IC_50_ value of 13.90 *µ*g/ml followed by BW2 with IC_50_ value of 15.34 *µ*g/ml and BW3 with IC_50_ value of 18.20 *µ*g/ml, respectively, against the standard acarbose of IC_50_ value of 10.35 *µ*g/ml.

### 3.4. *In Vivo* Studies

#### 3.4.1. Acute Toxicity Study

Dose in the range of 200–1500 mg/kg body of the body weight, selected on the basis of the acute toxicity series finding LD_0_ to LD_100,_ was used in acute toxicity study. For the synthetic compounds, the detailed dosing regimen is presented in Table 3.

For acute toxicity study test during the first 4 h each animal was observed for behavioral and general toxicity changes individually and regularly. Then for 3 days the observations were carried on a daily basis. In this toxicological study, the newly synthesized compounds were evaluated for any adverse or toxic effects, but no abnormal effects were observed. About 1000 mg was found to be the safe range of the drug. Lethal dose (LD_50_) of compounds was roughly 1000 mg/kg in mice. For mice given 1000 mg/kg of the body weight of all synthesized compounds no abnormality was observed in nasal or ocular systems, respiration, fur and skin, perspiration, urinary incontinence, defecation incontinence, salivation, piloerection, blood pressure, heart rate and CNS abnormalities such as gait, drowsiness, and ptosis convulsions. As dose of >300–2000 is categorized as category 4 due to organization for economic cooperation and development-(OECD) guidelines.

### 3.5. Antidiabetic Activity of the Synthesized Compounds in Diabetic Mice (By Alloxan)

In this assay, we tested three synthesized compounds based on *in vitro* antioxidant and antidiabetic activities. The BW1 exhibited decrease in blood glucose level over 15 days, i.e., 121, 207, 49, 34, and 33 mg/dl at concentration ranging from 500, 250, 125, 62.5, and 31.25 *µ*g/kg, respectively, while BW2 displayed a decrease of 199, 111, 68, 58, and 35 mg/dl of blood glucose level, respectively, while BW3 exhibited decrease in blood glucose levels of 49, 35, −29, −56 and −39 mg/dl. Out of the three synthesized compounds, BW1 displayed the highest antidiabetic potential as given in Table 4. Glibenclamide was used as positive control in this assay which displayed 198 mg/dl blood glucose level over 15 days.

### 3.6. Oral Glucose Tolerance Test

To perform OGTT, mice fasted overnight were used including both control and treated mice. Glucose was administered orally at the dose of 2 g/kg against glibenclamide as a standard drug. After administration of glucose, blood glucose level was calculated at time intervals of 0, 30, 60, and 120 min for assessment of the impact of exogenously given D-glucose on treated mice. BW1 treated mice showed good result of 160.3 mg/dl followed by BW2 (151.4 mg/dl) and BW3 (145.4 mg/dl) against the standard glibenclamide (140.5 mg/dl) after 120 minutes of glucose administration as evident from Table 5.

## 4. Discussion

DM is a group of metabolic disorders involving disturbances of carbohydrate, fat, and protein metabolism resulting from defects in insulin secretion, insulin action, or both. It is characterized by chronic hyperglycemia and other abnormalities including defect in insulin secretion, resistance to insulin in liver, skeletal muscles, and adipose tissues, and exaggerated hepatic glucose production. Glucosidase enzymes lead to the hydrolysis of complex carbohydrates to convert them into glucose for intestinal absorption. Two major types of carbohydrate-metabolizing enzymes are *α*-amylase and *α*-glucosidase. The *α*-amylases consist of salivary alpha amylase and pancreatic alpha amylase. Salivary alpha amylase convert starch into short oligomers through the cleavage of endo *α*-(1,4) linkages present in starch. Pancreatic alpha amylase hydrolyzes these oligomers to produce smaller oligosaccharides such as dextrin, maltotriose, and maltose [29].

The oligosaccharides produced by alpha amylase cannot undergo intestinal absorption, therefore, *α*-glucosidase such as maltase-glucoamylase and sucrase-isomaltase cleave *α*-(1,4) glycosidic linkages to produce dextrose [30]. Reduction of postprandial hyperglycemia can be obtained by inhibition of these carbohydrate-metabolizing enzymes [31]. The *α*-glucosidase inhibitors are distinctive drugs used for the management of diabetes. This class of drugs has no effect on pancreas. They control postprandial hyperglycemia by delaying the absorption of carbohydrates from GIT [32]. In the current study, we have explored the potential of newly synthesized compounds as *α*-glucosidase and *α*-amylase inhibitors.

OGTT is commonly used in clinics and in research [33] for the diagnosis of type 2 diabetes mellitus and impaired glucose tolerance [34]. It has also been used for the investigation of glucose sensitivity and glucose utilization in animals in research. In OGTT, the patient is given an oral bolus of glucose, and blood glucose levels are measured at specified intervals of time to measure the response of pancreatic beta cells and tolerance to glucose [35]. The OGTT is a shorter test in which over a period of 2 h three blood samples are taken for measurement of glucose levels [36]. This test was designed for simulation of physiological conditions and relation between Insulin secretion and glucose levels.

According to the recent research, ROS are produced at greater rate in diabetic patients, and it has been found that they are strongly implicated in the development of diabetes-associated complications [37]. This increase in ROS cause damage especially in the postprandial state where there is acute rise in blood glucose levels [13]. According to literature, ROS are produced in diabetes through different mechanisms including nonenzymatic glycosylation reaction, electron transport chain in mitochondria, and membrane-bound NADPH-oxidase. During persistent hyperglycemia, glucose forms covalent bond with proteins through nonenzymatic reaction leading to the production of glycated proteins such as glycated albumin and glycated hemoglobin. In diabetics, electron transport chain in mitochondria is activated to a greater extent than normal individuals, which leads to overproduction of ROS. Angiotensin II and advanced glycation end products induce the production of NADPH-oxidase that further increases ROS production [38]. These ROS are involved in pancreatic beta cell dysfunction, insulin resistance, and atherosclerotic complications of diabetes. Lower expression of glutathione and catalase antioxidant enzymes makes pancreatic beta cells more susceptible to ROS, which leads to beta cell dysfunction [39]. It has been cited in literature that chronic consumption of an antioxidant, alpha-lipoic acid reduced insulin resistance supporting the study that antioxidants are involved in decreasing insulin resistance [40]. These investigations suggest that the treatment of diabetes with potent antioxidant drugs would have significant effect on the development of DM2 and atherosclerosis in future. In the current study, ABTS and DPPH antioxidant assays were conducted for the synthesized compounds to investigate their antioxidant potential.

According to literature, several succinimide derivatives were found to have antidiabetic activities such as cyanoacetate derivatives [41], (S)-1-(2,5-dioxo-1-phenylpyrrolidin-3-yl) cyclohexanecarbaldehyde and (R)-2-((S)-2,5-dioxo-1-phenylpyrrolidin-3-yl)-2-phenylpropanal [42], and isothiocyanate derivatives of succinimides [43]. No studies were found on ketone derivatives of succinimides as antidiabetic potential. Based on the structure resemblance of the ketone derivatives with antidiabetic agents containing five membered rings such as thaizolidinedione, it is assumed that these derivatives have antidiabetic potential.

## 5. Conclusion

The identification and potential of some new succinimide derivatives is reported herein. All the ketone derivatives of succinimide displayed significant inhibitory potential. The synthesized compounds showed better ABTS and DPPH scavenging activities. Apparently, BW1 showed better inhibition of alpha amylase and alpha glucosidase enzymes. In the *in vivo* acute toxicity study, no unusual signs were observed. Moreover, we also subjected these three compounds (BW1, BW2, and BW3) to the *in vivo* studies. Our compounds showed significant hypoglycemic effects as compared to the standard drug glibenclamide. These compounds were not assessed previously for their antioxidant and antidiabetic potential. In future work, we planned to use medicinal chemistry approaches to design succinimide conjugates with ester as bioactive scaffolds.

## Figures and Tables

**Table 1:** The percent inhibition of ABTS and DPPH of synthesized compounds.

Compound	Conc. (*µ*g/ml)	(%) ABTS inhibitions	IC_50_ (*μ*g/ml)	(%) DPPH inhibitions	IC_50_ (*μ*g/ml)
BW1	500	78.35 ± 0.23^*∗∗∗*^	9.40	85.83 ± 0.47^*∗∗*^	10.84
	250	73.36 ± 0.84^*∗∗∗*^		77.23 ± 0.96^*∗∗∗*^	
	125	70.62 ± 0.25^*∗∗∗*^		67.33 ± 0.55^*∗∗∗*^	
	62.50	66.16 ± 0.16^*∗∗∗*^		72.29 ± 0.57^*∗∗∗*^	
	31.25	55.67 ± 0.32^*∗∗∗*^		62.03 ± 0.77^*∗∗∗*^	

BW2	500	76.85 ± 2.24^*∗∗∗*^	12.87	75.09 ± 0.32^*∗∗∗*^	12.81
	250	71.08 ± 0.47^*∗∗∗*^		71.67 ± 1.20^*∗∗∗*^	
	125	66.90 ± 0.96^*∗∗∗*^		66.40 ± 0.25^*∗∗∗*^	
	62.50	61.35 ± 0.51^*∗∗∗*^		61.58 ± 1.12^*∗∗∗*^	
	31.25	57.40 ± 0.76^*∗∗∗*^		56.65 ± 1.34^*∗∗∗*^	

BW3	500	70.56 ± 1.06^*∗∗∗*^	36.82	83.03 ± 0.48^*∗*^	15.93
	250	64.90 ± 0.45^*∗∗∗*^		76.90 ± 0.48^*∗*^	
	125	58.40 ± 0.82^*∗∗∗*^		71.79 ± 0.63^*∗∗*^	
	62.50	53.33 ± 0.66^*∗*^		66.67 ± 0.61^*∗∗∗*^	
	31.25	48.42 ± 0.43^ns^		57.69 ± 0.77^*∗∗∗*^	

Ascorbic acid	500	87.08 ± 0.47	6.84	90.65 ± 1.32	7.10
	250	82.40 ± 0.20		84.56 ± 0.45	
	125	77.61 ± 0.43		79.52 ± 0.66	
	62.50	75.45 ± 0.90		73.22 ± 1.28	
	31.25	63.89 ± 0.20		68.42 ± 0.43	

All values are taken as mean ± SEM (*n* = 3), and two-way ANOVA followed by bonferroni test were followed. Values are significantly different as compared to the standard drug; ^*∗*^*P* < 0.05, ^*∗∗*^*P* < 0.01, ^*∗∗∗*^*P* < 0.001, and ns: not significant.

**Table 2:** The percent inhibition of alpha-amylase and alpha-glucosidase of the synthesized compounds.

Compound	Conc. (*µ*g/ml)	% inhibitions (*α*-glucosidase)	IC_50_ (*μ*g/ml)	% inhibitions (*α*-amylase)	IC_50_ (*μ*g/ml)
BW1	500	89.08 ± 1.04^ns^	10.49	81.60 ± 0.00^*∗∗∗*^	13.90
	250	83.45 ± 0.90^ns^		75.32 ± 0.40^*∗∗∗*^	
	125	76.58 ± 0.63^ns^		71.78 ± 0.44^*∗∗∗*^	
	62.50	70.40 ± 0.20^ns^		65.08 ± 0.66^*∗∗∗*^	
	31.25	65.80 ± 0.90^ns^		57.40 ± 0.40^*∗∗∗*^	

BW2	500	87.36 ± 0.49^*∗*^	10.81	78.69 ± 0.14^*∗∗∗*^	15.34
	250	81.34 ± 0.55^*∗*^		73.14 ± 0.49^*∗∗∗*^	
	125	74.39 ± 0.49^*∗∗∗*^		67.44 ± 0.15^*∗∗∗*^	
	62.50	68.47 ± 0.52^*∗∗∗*^		63.72 ± 0.11^*∗∗∗*^	
	31.25	62.44 ± 0.55^*∗∗*^		55.85 ± 0.17^*∗∗∗*^	

BW3	500	77.85 ± 2.24^*∗∗∗*^	12.15	75.35 ± 0.89^*∗∗∗*^	18.20
	250	72.08 ± 0.47^*∗∗∗*^		71.36 ± 1.15^*∗∗∗*^	
	125	67.90 ± 0.96^*∗∗∗*^		67.62 ± 0.03^*∗∗∗*^	
	62.50	63.28 ± 0.57^*∗∗∗*^		62.16 ± 0.12^*∗∗∗*^	
	31.25	57.47 ± 0.56^*∗∗∗*^		54.67 ± 0.35^*∗∗∗*^	

Acarbose	500	92.23 ± 0.22	8.20	87.75 ± 0.42	10.35
	250	86.45 ± 0.90		82.47 ± 0.71	
	125	80.90 ± 0.60		76.20 ± 0.49	
	62.50	73.00 ± 0.30		69.42 ± 1.55	
	31.25	67.90 ± 0.45		63.62 ± 0.58	

All values are taken as mean ± SEM (*n* = 3), and two-way ANOVA followed by Bonferroni test were followed. Values significantly different as compared to standard drug; ^*∗*^*P* < 0.05, ^*∗∗*^*P* < 0.01, ^*∗∗∗*^*P* < 0.001, and ns: not significant.

**Table 3:** Acute toxicity studies with tested synthesized compounds.

Groups	Animals	Tested compounds (mg/kg) BW1, BW2, BW3
1	6	200
2	6	300
3	6	400
4	6	500
5	6	1000
6	6	1500

*n* = 6 per group.

**Table 4:** *In vivo* results of synthesized compounds against the standard drug.

S.no	Groups		Dose *μ*g/kg	Blood glucose level (mg/dl)					Decrease in blood glucose after 15 days (mg/dl)	Change in body weight (Gm)
				0 day	4^th^ day	7^th^ day	10^th^ day	15^th^ day		
1	Diabetic control		0.35 ml	478	483	501	514	527	−49	−13.7
2	Normal control saline		0.35	125	111	103	96	91	34	
3	Glibenclamide		0.5	473	303	257	212	198	275	+10.3

4	BW1	1	500	412	388	372	312	291	121	+7.1
		2	250	421	397	345	219	214	207	+5.7
		3	125	443	431	413	401	394	49	+4.1
		4	62.5	380	371	365	351	346	34	+4.3
		5	31.25	418	408	399	382	375	33	+2.3

5	BW2	1	500	456	402	361	303	257	199	+7.4
		2	250	466	461	443	407	355	111	+3.4
		3	125	437	431	422	394	369	68	−3.7
		4	62.5	386	359	349	336	328	58	−4.6
		5	31.25	452	448	431	425	417	35	−6.8

6	BW3	1	500	430	420	401	387	381	49	+2.5
		2	250	431	419	411	403	396	35	−5.1
		3	125	387	437	430	424	416	−29	+5.3
		4	62.5	443	465	478	493	499	−56	−6.2
		5	31.25	439	446	457	468	478	−39	−8.4

**Table 5:** Oral glucose tolerance test results.

Treatment	Conc/route	OGTT (mg/dl)			
		0 minutes	30 minutes	60 minutes	120 minutes
Group-I (Tween80)	Oral	212.9	230.3	252.7	295.3
Group-II (GB)	Oral	151.5	176.7	214.3	140.5
BW1	Oral	165.8	194.4	211.8	160.3
BW2	Oral	163.7	189.2	214.8	151.4
BW3	Oral	156.2	174.1	196.3	145.4

## Data Availability

All data used to support the findings of this study are included within the article.

## References

[1] Buowari O. Y. (2013). Diabetes mellitus in developing countries and case series. *Diabetes Mellitus-Insights And Perspectives*.

[2] Wild S., Roglic G., Green A., Sicree R., King H. (2004). Global prevalence of diabetes. *Diabetes Care*.

[3] Pavithra D., Praveen D., Chowdary P. R., Aanandhi M. V. (2018). A review on role of vitamin E supplementation in type 2 diabetes mellitus. *Drug Invention Today*.

[4] American Diabetes Association (2014). Diagnosis and classification of diabetes mellitus. *Diabetes Care*.

[5] Galicia-Garcia U., Benito-Vicente A., Jebari S. (2020). Pathophysiology of type 2 diabetes mellitus. *International Journal of Molecular Sciences*.

[6] Poongunran J., Perera H., Fernando W., Jayasinghe L., Sivakanesan R. (2015). *α*-glucosidase and *α*-amylase inhibitory activities of nine Sri Lankan antidiabetic plants. *British Journal of Pharmaceutical Research*.

[7] Nair S. S., Kavrekar V., Mishra A. (2013). In vitro studies on alpha amylase and alpha glucosidase inhibitory activities of selected plant extracts. *European Journal of Experimental Biology*.

[8] Tayab M. A., Chowdhury K. A. A., Jabed M. (2021). Antioxidant-rich woodfordia fruticosa leaf extract alleviates depressive-like behaviors and impede hyperglycemia. *Plants*.

[9] Papatheodorou K., Papanas N., Banach M., Papazoglou D., Edmonds M. (2016). Complications of diabetes 2016. *Journal of Diabetes Research*.

[10] Keenan T. D. L., Johnston R. L., Donachie P. H. J., Sparrow J. M., Stratton I. M., Scanlon P. (2013). United Kingdom national ophthalmology database study: diabetic retinopathy; report 1: prevalence of centre-involving diabetic macular oedema and other grades of maculopathy and retinopathy in hospital eye services. *Eye*.

[11] Leustean A. M., Ciocoiu M., Sava A. (2018). Implications of the intestinal microbiota in diagnosing the progression of diabetes and the presence of cardiovascular complications. *Journal of Diabetes Research*.

[12] Zafar R., Zubair M., Ali S. (2021). Zinc metal carboxylates as potential anti-alzheimer’s candidate: in vitro anticholinesterase, antioxidant and molecular docking studies. *Journal of Biomolecular Structure and Dynamics*.

[13] Piconi L., Quagliaro L., Ceriello A. (2003). Oxidative stress in diabetes. *Clinical Chemistry and Laboratory Medicine*.

[14] Ceriello A. (2003). New insights on oxidative stress and diabetic complications may lead to a “causal” antioxidant therapy. *Diabetes Care*.

[15] Islam M. N., Tasnim H., Arshad L. (2020). Stem extract of *Albizia richardiana* exhibits potent antioxidant, cytotoxic, antimicrobial, anti-inflammatory and thrombolytic effects through in vitro approach. *Clinical Phytoscience*.

[16] Eruygur N., Esra U. (2018). Cholinesterase, *α*-glucosidase, *α*-amylase, and tyrosinase inhibitory effects and antioxidant activity of veronica officinalis extracts. *Türkiye Tarımsal Araştırmalar Dergisi*.

[17] Triggle C. R., Ding H. (2014). Cardiovascular impact of drugs used in the treatment of diabetes. *Therapeutic Advances In Chronic Disease*.

[18] Kavitha K., Sri Shanthi Praveena K., Veera Venkat Shivaji Ramarao E., Yadagiri Sreenivasa Murthy N., Pal S. (2016). Chemistry of cyclic imides: an overview on the past, present and future. *Current Organic Chemistry*.

[19] Sadiq A., Mahnashi M. H., Alyami B. A., Alqahtani Y. S., Alqarni A. O., Rashid U. (2021). Tailoring the substitution pattern of Pyrrolidine-2,5-dione for discovery of new structural template for dual COX/LOX inhibition. *Bio Organic Chemistry*.

[20] Zhang Y., Wang W. (2012). Recent advances in organocatalytic asymmetric michael reactions. *Catalysis Science and Technology*.

[21] Sadiq A., Nugent T. C. (2020). Catalytic access to succinimide products containing stereogenic quaternary carbons. *Chemistry Select*.

[22] Jan M. S., Ahmad S., Hussain F. (2020). Design, synthesis, in-vitro, in-vivo and in-silico studies of pyrrolidine-2,5-dione derivatives as multitarget anti-inflammatory agents. *European Journal of Medicinal Chemistry*.

[23] Re R., Pellegrini N., Proteggente A., Pannala A., Yang M., Rice-Evans C. (1999). Antioxidant activity applying an improved ABTS radical cation decolorization assay. *Free Radical Biology & Medicine*.

[24] Miser-Salihoglu E., Akaydin G., Caliskan-Can E., Yardim-Akaydin S. (2013). Evalution of antioxidant activity of various herbal folk medicines. *Nutrition & Food Science*.

[25] Yao X., Zhu L., Chen Y., Tian J., Wang Y. (2013). In vivo and in vitro antioxidant activity and *α*-glucosidase, *α*-amylase inhibitory effects of flavonoids from cichorium glandulosum seeds. *Food Chemistry*.

[26] Kinsner-Ovaskainen A., Rzepka R., Rudowski R., Coecke S., Cole T., Prieto P. (2009). Acutoxbase, an innovative database for in vitro acute toxicity studies. *Toxicology in Vitro*.

[27] Barkat M. A., Mujeeb M. (2013). The comparative study of catharanthus roseus extract and extract loaded chitosan nanoparticles in alloxan induced diabetic rats. *International Journal of Biomedical Research*.

[28] Smith N. (2020). Oral glucose tolerance test in mouse. *Protocols io*.

[29] Sadiq A. (2020). Treating hyperglycemia from Eryngium caeruleum M. Bieb: in-vitro *α*-glucosidase, antioxidant, in-vivo antidiabetic and molecular docking-based approaches. *Frontiers in Chemistry*.

[30] Amiri M., Naim H. Y. (2018). Posttranslational processing and function of mucosal maltases. *Journal of Pediatric Gastroenterology and Nutrition*.

[31] Mahomoodally M. F., Subratty A. H., Gurib-Fakim A., Choudhary M. I., Nahar Khan S. (2012). Traditional medicinal herbs and food plants have the potential to inhibit key carbohydrate hydrolyzing enzymes in vitro and reduce postprandial blood glucose peaks in vivo. *The Scientific World Journal*.

[32] Kalra S. (2014). Alpha glucosidase inhibitors. *JPMA. The Journal of the Pakistan Medical Association*.

[33] Kuo F. Y., Cheng K.-C., Li Y., Cheng J.-T. (2021). Oral glucose tolerance test in diabetes, the old method revisited. *World Journal of Diabetes*.

[34] Sekikawa A., Eguchi H., Tominaga M. (2000). Prevalence of type 2 diabetes mellitus and impaired glucose tolerance in a rural area of Japan. *Journal of Diabetes and Its Complications*.

[35] Ionescu E., Sauter J. F., Jeanrenaud B. (1985). Abnormal oral glucose tolerance in genetically obese (fa/fa) rats. *American Journal of Physiology-Endocrinology And Metabolism*.

[36] Phillips D. I. W., Clark P. M., Hales C. N., Osmond C. (1994). Understanding oral glucose tolerance: comparison of glucose or insulin measurements during the oral glucose tolerance test with specific measurements of insulin resistance and insulin secretion. *Diabetic Medicine*.

[37] Islam M. N., Rauf A., Fahad F. I. (2021). Superoxide dismutase: an updated review on its health benefits and industrial applications. *Critical Reviews in Food Science and Nutrition*.

[38] Kaneto H., Katakami N., Matsuhisa M., Matsuoka T. A. (2010). Role of reactive oxygen species in the progression of type 2 diabetes and atherosclerosis. *Mediators of Inflammation*.

[39] Karunakaran U., Park K.-G. (2013). A systematic review of oxidative stress and safety of antioxidants in diabetes: focus on islets and their defense. *Diabetes & metabolism journal*.

[40] Ansar H., Mazloom Z., Kazemi F., Hejazi N. (2011). Effect of alpha-lipoic acid on blood glucose, insulin resistance and glutathione peroxidase of type 2 diabetic patients. *Saudi Medical Journal*.

[41] Hussain F., Khan Z., Jan M. S. (2019). Synthesis, in-vitro *α*-glucosidase inhibition, antioxidant, in-vivo antidiabetic and molecular docking studies of pyrrolidine-2,5-dione and thiazolidine-2,4-dione derivatives. *Bio Organic Chemistry*.

[42] Ahmad A., Ullah F., Sadiq A. (2020). Comparative cholinesterase, *α*-glucosidase inhibitory, antioxidant, molecular docking, and kinetic studies on potent succinimide derivatives. *Drug Design, Development and Therapy*.

[43] Yılmaz F., Menteşe E., Baltaş N. (2019). Synthesis and biological evaluation of some succinimide hybrid molecules. *Russian Journal of Bioorganic Chemistry*.

